# Physics-informed neural ODE (PINODE): embedding physics into models using collocation points

**DOI:** 10.1038/s41598-023-36799-6

**Published:** 2023-06-22

**Authors:** Aleksei Sholokhov, Yuying Liu, Hassan Mansour, Saleh Nabi

**Affiliations:** 1 Department of Applied Mathematics, University of Washington, Seattle, USA; 2 Mitsubishi Electric Research Laboratories, Cambridge, 02139 USA

**Keywords:** Computational science, Applied mathematics, Computer science

## Abstract

Building reduced-order models (ROMs) is essential for efficient forecasting and control of complex dynamical systems. Recently, autoencoder-based methods for building such models have gained significant traction, but their demand for data limits their use when the data is scarce and expensive. We propose aiding a model’s training with the knowledge of physics using a collocation-based physics-informed loss term. Our innovation builds on ideas from classical collocation methods of numerical analysis to embed knowledge from a known equation into the latent-space dynamics of a ROM. We show that the addition of our physics-informed loss allows for exceptional data supply strategies that improves the performance of ROMs in data-scarce settings, where training high-quality data-driven models is impossible. Namely, for a problem of modeling a high-dimensional nonlinear PDE, our experiments show $\times$ 5 performance gains, measured by prediction error, in a low-data regime, $\times$ 10 performance gains in tasks of high-noise learning, $\times$ 100 gains in the efficiency of utilizing the latent-space dimension, and $\times$ 200 gains in tasks of far-out out-of-distribution forecasting relative to purely data-driven models. These improvements pave the way for broader adoption of network-based physics-informed ROMs in compressive sensing and control applications.

## Introduction

Forecasting the behavior of a large-scale real-world system directly from first principles often requires solving highly-nonlinear governing equations such as high-dimensional ordinary differential equations (ODEs) or partial differential equations (PDEs). High-fidelity simulations of such dynamical systems can become intractable, especially if an online control algorithm requires multiple forecasts per second using a low-powered embedded device^1–3^. A situation like this arises, for example, when a smart heating, ventilation, and air conditioning (HVAC) system attempts to optimize the temperature distribution of the air in a room using only partial measurements^4,5^. At the time of writing this paper, such systems are incapable of real-time complex simulations, but they can already run low-dimensional pre-trained models, which invites the development of high-quality reduced order models (ROMs)^6^. Therefore, ROMs are essential for enabling the design optimization, uncertainty propagation, predictive modeling, and control for such dynamical systems^1,7–9^

In order to enable control of high dimensional dynamical systems, a ROM training method needs to identify a low-dimensional manifold along with dynamics on the manifold that together yield high-accuracy predictions and long-term stability^10,11^. Most traditional ROMs are projection-based, e.g. dynamic mode decomposition (DMD)^8,12^ and proper orthogonal decomposition (POD)^13^, which transform the trajectories of a high-dimensional dynamical system into a suitable, and in some sense optimal, low-dimensional subspace. This projection leads to truncation of higher order modes and parametric uncertainties, which result in large prediction errors over time due to the deterioration of the basis functions (spatial modes)^3^. One challenge for POD methods is their intrusive nature, i.e. requiring access to the solver codes. To overcome this, operator inference approaches^14,15^ utilize SVD-based model reduction and exploit lifting to fit the latent space dynamics data into polynomial, typically quadratic, models. These models, however, are (i) limited in representation power (up to quadratic, e.g. for lift and learn approach) and (ii) require a custom-tailored SVD-based optimization technique.

In a thrust to overcome these challenges, significant effort has been invested into developing autoencoder-based reduced-order models, as a popular nonlinear ROM technique, which can yield both accurate and stable ROMs^16–19^. In practice, however, autoencoder-based ROMs require datasets that densely cover a hypothetical infinite dimensional phase portrait of the dynamical system. Moreover, the large demand for training data significantly limits the use of such models in physics applications where the data can be expensive to obtain.

Another severe challenge of utilizing ROMs comes from their poor out-of-distribution performance^17,20,21^, especially when it is fundamentally impossible for a practitioner to obtain data that covers the entire distribution of possible data inputs. For example, in HVAC applications, one may collect data from a room with two windows but not from a room for every possible number of windows. In atmospheric LiDAR applications, we may conduct experiments on a certain terrain but we can never conduct experiments on all sorts of terrains^22^. In such situations embedding the knowledge of physics into a model becomes necessary to improve the extrapolation performance, and for which several approaches have recently been proposed. For instance, the seminal works^23,24^ have tried to determine the underlying structure of a nonlinear dynamical system from data using symbolic regression. Recently, Cranmer et al.^21^ employed symbolic regression in conjunction with graph neural network (GNN), while encouraging sparse latent representation, to extract explicit physical relations. They showed that the symbolic expressions extracted from the GNN generalized to out-of-distribution data better than the GNN itself. However, symbolic regression also suffers from excessive computational costs, and may be prone to overfitting.

Another example of incorporating physics in ROMs is the use of parametric models at the latent space, e.g. by using the sparse identification of nonlinear dynamics (SINDy)^18,25^. For instance,^20,26^ used a chain-rule based loss that ties latent-space derivatives to the observable-space derivatives for simultaneous training of the autoencoder and the latent dynamics. However, such loss is highly sensitive to noise in the data, especially when evaluating time-derivatives with finite differences is required^27^. Collocation-based enforcement of the physics, i.e. projection of the candidate functions in the governing equations to enforce the chain rule instead of finite difference, could address such numerical difficulties. Recently, Liu et al.^28^ used an autoencoder architecture and Koopman theory to demonstrate that combining autoencoders with enforcing linear dynamics in the latent space may result in an interpretable ROM. However, linearity may not be expressive enough for complex dynamics with multiple basins of attraction^29^. Finally, recent works on NeuralODE (NODE)^30,31^ show a way to fit an arbitrary non-linear model (e.g. a network) as a latent space dynamics model, significantly extending the set of models for the latent dynamics that one can train efficiently.

In this paper, we employ autoencoders to perform nonlinear model reduction along with NODE in the latent space to model complex and nonlinear dynamics. We choose Neural ODEs in the latent space dynamics representation because of their ability to model highly non-linear dynamics, which is especially important when applications limit the size of the latent space dimension. Our goal is to reduce the demand for training data and improve the overall forecasting stability under challenging training conditions. To that end, we build on ideas from classical collocation methods of numerical analysis to embed knowledge from a known governing equation into the latent-space dynamics of a ROM, as described in “Methods” section. In “Experiments” section, we show that the addition of our physics-informed loss allows for exceptional data supply strategies that improves the performance of ROMs in data-scarce settings, where training high-quality data-driven models is impossible. We demonstrate that such an approach not only reduces the need for large training data-sets and produces highly-accurate and long-term stable models, but also leads to the discovery of more compact latent spaces, which is especially important for applications in compressed sensing and control.

## Methods

### Reduced-order model with non-linear latent dynamics

We consider an autonomous dynamical system on a finite space $\mathscr {X}\subseteq {\mathbb {R}}^n$ given by

1 $$\begin{aligned} \frac{\text{d}}{\text{d}t}\varvec{x}(t) = \varvec{f}(\varvec{x}(t)). \end{aligned}$$

In real-world applications, it is often expensive to solve Eq. (1) directly because *x*(*t*) can be very high-dimensional. However, a variety of works provided both theoretical^13^ and empirical^11,32^ evidence that many physical systems evolve on a manifold $\mathscr {Z}\subseteq {\mathbb {R}}^m$ of a lower dimension $m<< n$. In that space, the dynamics evolve according to a (generally unknown) function $\varvec{h}(\varvec{z})$:

2 $$\begin{aligned} \frac{\text{d}}{\text{d}t}\varvec{z}(t) = \varvec{h}(\varvec{z}(t)). \end{aligned}$$

We call the space $\mathscr {X}$ an observable space, and $\mathscr {Z}$ a latent space. When an invertible mapping $\psi :\ \mathscr {Z}\rightarrow \mathscr {X}$ between the observable and the latent spaces is known, one can predict the dynamics of the system $\varvec{x}$ at a future time *T* by projecting the initial condition $\varvec{x}(0)$ into the latent space, integrating the dynamics in the latent space, and mapping the resulting trajectory back to the observable space:

3 $$\begin{aligned} \begin{aligned} \varvec{z}(0)&= \psi ^{-1}(\varvec{x}(0)) \\ \varvec{z}(T)&= \varvec{z}(0) + \int _{0}^T\varvec{h}(\varvec{z}(t))dt \\ \varvec{x}(T)&= \psi (\varvec{z}(T)). \end{aligned} \end{aligned}$$

When $m<< n$ we refer to the triplet $(\psi , \psi ^{-1}, \varvec{h})$ as a Reduced-Order Model (ROM) of $\varvec{f}$. It is often the case that for a given system $\varvec{f}$, there exists no ROM $(\psi , \psi ^{-1}, \varvec{h})$ such that the relation (3) holds exactly. In this case, we seek an *approximation* ROM $(\psi _{\theta ^*}, \phi _{\theta ^*}, h_{\theta ^*})$ that minimizes the difference between the data *x*(*t*) and the prediction ${\hat{x}}(t)$ over a chosen class of models $(\psi _\theta , \phi _\theta , h_\theta )$ parameterized by $\theta$.

Multiple real-world applications necessitate using ROMs instead of integrating the relation (1) directly. For example, integrating (1) may be computationally intractable especially on platforms with limited computing capability such as embedded and autonomous devices. For instance, in an HVAC system, solving (1) means solving a Navier–Stokes equation on a fine grid in real time, which exceeds the computing capabilities of current-generation appliances. On the other hand, integrating (3) may be cheap when $m<< n$. Finally, even when solving (1) is possible in real time (e.g. by utilizing a remote cluster), executing control over the resulting model, which is an end-goal for an HVAC system, may still be intractable. Indeed, executing control requires *multiple* evaluations of (1) for *each* iteration of control even for the most efficient algorithms known to date^33^.

**Figure 1:**
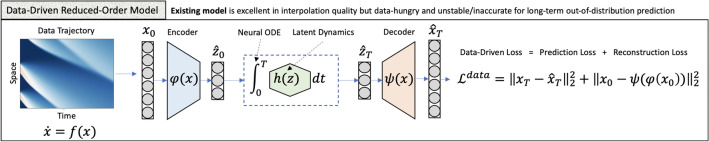
Illustration of the autoencoer structure with neural ODE in the latent space. The data-driven part of the loss function aims to minimize a sum of two objectives: the prediction loss and the reconstruction loss. The prediction loss minimizes the difference between the data trajectories and their model predictions to ensure temporal consistency of the latent space dynamics. The reconstruction loss ensures accurate reconstruction of individual snapshots, ensuring that the autoencoder behaves as an invertible mapping on all snapshots.

### Architecture

In this work we model $\psi$, $\psi ^{-1}$, and $\varvec{h}$ with fully-connected neural networks $\psi _\theta$, $\phi _\theta$, and $h_\theta$, respectively. Specifically, the pair ($\psi$, $\psi ^{-1}$) is modelled with an autoencoder $(\psi _\theta , \phi _\theta )$, and $\varvec{h}$ is modelled with a fully-connected network $h_\theta$. Figure 1 visualizes the architecture of the model.

### Data-driven loss

Similar to prior works^17,34,35^, we define a *data-driven loss* $\mathscr {L}_{data}$ as a sum of reconstruction and prediction losses. The former ensures that $\phi _\theta$ and $\psi _\theta$ are inverse mappings of each other, whereas the latter matches the model’s predictions to the available data, as illustrated on Fig. 1.

Formally, for a given set of trajectories $\varvec{x}_i$, $i \in [1 \dots k]$, where each trajectory $\varvec{x}_i \in {\mathbb {R}}^{n \times p}$ is a set of *p* snapshots that correspond to the recorded states of the system for *p* time-steps, $t_j$, $j \in [1, \dots , p]$, the loss function $\mathscr {L}^{data}_\theta$ is defined as:

4 $$\begin{aligned} \mathscr {L}^{data}_\theta&= \frac{1}{2\sigma ^2}\sum _{i = 1}^k \left[ \frac{\omega _1}{p}\sum _{j=1}^p\left\| \varvec{x_i}(t_j) - \psi _\theta (\phi _\theta (\varvec{x_i}(t_j)))\right\| ^2\right. +, \end{aligned}$$

5 $$\begin{aligned}&+ \left. \frac{\omega _2}{p}\sum _{j=1}^p \left\| \psi _\theta \left( \phi _\theta (\varvec{x_i}(t_1)) + \int _{t_1}^{t_j}h(z(t))dt\right) - \varvec{x_i}(t_j)\right\| ^2 \right] , \end{aligned}$$

where $\sigma$ is the standard deviation of the observation noise. We note that each trajectory $\varvec{x}_i$ may be captured over its own time-frame and may use a distinct, possibly non-uniform, step-size, in which case the loss function should be modified accordingly [The implementation is affected only in evaluating the integral in (4). This part is handled by torchdiffeq^36^ library, which supports non-uniform time-frames within a batch]. To simplify the notation, without loss of generality, in the rest of the paper we assume that all trajectories are recorded over the same time-frame with the same uniform step-size. To forecast the behavior of the system in the latent space, we apply the technique of Neural Ordinary Differential Equations (Neural ODEs or NODEs)^30^, which utilizes the adjoint sensitivity method to back-propagate the gradients through the integral in (4). Neural ODEs have demonstrated a better ability to model highly non-linear dynamics compared to linear models when the dimensionality of the dynamics variable is limited. This is especially useful in applications where the size of the latent space dimension needs to be small^16–19^.

### Physics-informed loss

In their recent work, Liu et al.^28^ proposed a method for utilizing knowledge of the governing equations $d\varvec{x}/dt = \varvec{f(x)}$ as a finite-dimensional approximation of Koopman eigenfunctions for linear latent dynamics. To extend this approach to the non-linear regime, we note that for a true mapping $\phi$ the following holds:

6 $$\begin{aligned} \frac{d\varvec{z}(\varvec{x}(t))}{dt} = \frac{d\varvec{z}}{d\varvec{x}}\frac{d\varvec{x}}{dt} = \nabla \phi (\varvec{x}(t))^T\varvec{f}(\varvec{x(t)}). \end{aligned}$$

On the other hand, by the definition of $\psi$ and $\varvec{h}$ we have that

7 $$\begin{aligned} \frac{d\varvec{z}(\varvec{x}(t))}{dt} = \varvec{h}(\phi (\varvec{x}(t)). \end{aligned}$$

Combining Eqs. (6) and (7) we get that

8 $$\begin{aligned} \varvec{h}(\phi (\varvec{x}(t)) = \nabla \phi (\varvec{x})^T\varvec{f}(\varvec{x}). \end{aligned}$$

**Figure 2:**
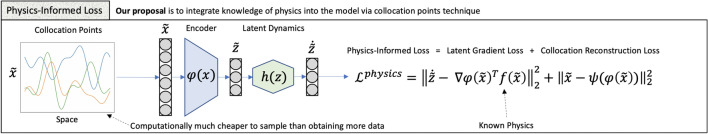
The physics-informed loss function compares gradient fields in the current latent space with what a correctly-learned field should be in this latent space on set of collocation points.

Equation (8) links the dynamics $\varvec{h}(\varvec{z})$ and the encoder $\phi (\varvec{x})$ with the known equation $\varvec{f}(\varvec{x})$ and is true for all $z \in \mathscr {Z}$ and $x \in \mathscr {X}$. Hence, as shown on Fig. 2, knowledge of $\varvec{f}$ can be assimilated into the model by evaluating Eq. (8) on a set of *N* carefully sampled points $\bar{\varvec{x}}_i \in \mathscr {X}$, $i \in [1, \dots , N]$:

9 $$\begin{aligned} \mathscr {L}^{physics}_\theta = \sum _{i = 1}^N \left[ \frac{\omega _3}{N}\left\| h_\theta (\phi _\theta (\bar{\varvec{x}}_i)) - \nabla \phi _\theta (\bar{\varvec{x}}_i) \varvec{f}(\bar{\varvec{x}_i})\right\| ^2 + \frac{\omega _4}{N}\left\| \bar{\varvec{x}}_i - \psi _\theta (\phi _\theta (\bar{\varvec{x}}_i))\right\| \right] . \end{aligned}$$

We refer to the points $\bar{\varvec{x}}_i$ as *collocation points*.

### Collocation points

We define a collocation as pair $(\varvec{\bar{x}},\, \varvec{f}(\varvec{\bar{x}}))$. collocation points are samples from the space $\mathscr {X}\times Im_{f}(\mathscr {X})$, and they should satisfy three conditions, ordered by importance:

1. *Simplicity* $\varvec{f}(\bar{\varvec{x}}_j)$ should be computationally cheap to evaluate. It is especially important for PDE systems, where $\varvec{f}$ may involve high-order derivatives.
2. *Representativeness* $\bar{\varvec{x}}_j$ should cover the space of states where one aims to improve the model’s performance or stability. Collocation points that a model might encounter and that are not represented by data snapshots are the best candidates.
3. *Feasibility* $\bar{\varvec{x}}_j \in \mathscr {X}$. In other words, $x_j$ should be an attainable state of the system. Collocation points outside of $\mathscr {X}$ may downgrade the performance of the autoencoder by forcing it to be an invertible function on a domain outside of $\mathscr {X}$.

Thus, an optimal sampling procedure for collocation points $\varvec{\bar{x}}_j$ is domain-specific and should be designed given a particular system $\varvec{f}$ and available data $\varvec{x}_i$. We show examples of how these conditions can be implemented for real systems in the following sections.

The above definition of collocation points is not to be confused with a classic notion of collocation points for finding numerical solutions for differential equations^37,38^. The classic notion refers to a set of points in time $[t_0, t_0 + c_1h, t_0 + c_2h, \dots , t_0 + h]$, $0< c1< c2< \dots < 1$ which are chosen to obtain an optimal local interpolant of a solution of a differential equation for a time-period between $t_0$ and $t_0 + h$. For example, *s* collocation points for Runge-Kutta methods are defined to provide an optimal Gauss-Legendre interpolant of order *s*; the coefficients $c_1, \dots , c_s$ come from a respective Butcher table. In contrast, we define collocation points as pairs $(\varvec{\bar{x}},\, \varvec{f}(\varvec{\bar{x}}))$ which are examples of mapping $x \rightarrow f(x)$. Our definition is built around solving an *inverse* problem of approximating $\dot{x} = f(x)$ with $f_\theta (x)$ and follows a recent work^28^ which develops upon a definition from Ref.^39^ with the difference being the sample space: instead of sampling from the spatiotemporal domain we sample them from an appropriate function space.

### Combined loss function

We train the model by optimizing a sum of the physics-informed loss (9) and the data-driven loss (4):

10 $$\begin{aligned} \min _\theta \left[ \mathscr {L}^{physics}_\theta + \mathscr {L}^{data}_\theta \right] . \end{aligned}$$

When $\omega _1 = \omega _2 = 0$ we have $\mathscr {L}^{data}_\theta = 0$, so we say that the model is (purely) *Physics-Informed*. Similarly, when $\omega _3 = \omega _4 = 0$ we have $\mathscr {L}^{physics}_\theta = 0$ and we say that the model is (purely) *Data-Driven*. When $\omega _i \ne 0, \, \forall i$, we say that the model is *Hybrid*.

The coefficients $\omega _i$ are hyper-parameters which need to be tuned using a validation dataset. However, in all experiments of this paper we set $\omega _i$ to be either 0 or 1, and we balance $\mathscr {L}^{physics}_\theta$ and $\mathscr {L}^{data}_\theta$ the choice of samples in a batch of training data. Specifically, we set the number of collocation points per batch $N_{batch}$ to be equal to the number of trajectories per batch $k_{batch}$ times the number of time-steps*T*: $N_{batch} = Tk_{batch}$. In this way both $\mathscr {L}^{physics}_\theta$ and $\mathscr {L}^{data}_\theta$ represent the loss for $Tk_{batch}$ snapshots of the system, providing on average a similar contribution of information to the overall loss function. More laborious approaches of hyper-parameter tuning did not yield sufficient systematic advantage to justify the labour compared to this simple strategy.

We use a pytorch^40^ implementation of the Adam algorithm^41^ for optimization. To evaluate $\nabla _\theta \mathscr {L}^{physics}_\theta$ and $\nabla _\theta \mathscr {L}^{data}_\theta$ we use torchdiffeq^36^ – a pytorch-compatible implementation of the Neural ODE framework.

To the best of our knowledge, this is the first framework that combines non-linear latent-dynamics (Neural ODE), autoencoders, and a physics-informed loss term (9). Thus, we call our framework *Physics-Informed Neural ODE*, or PINODE.

**Figure 3:**
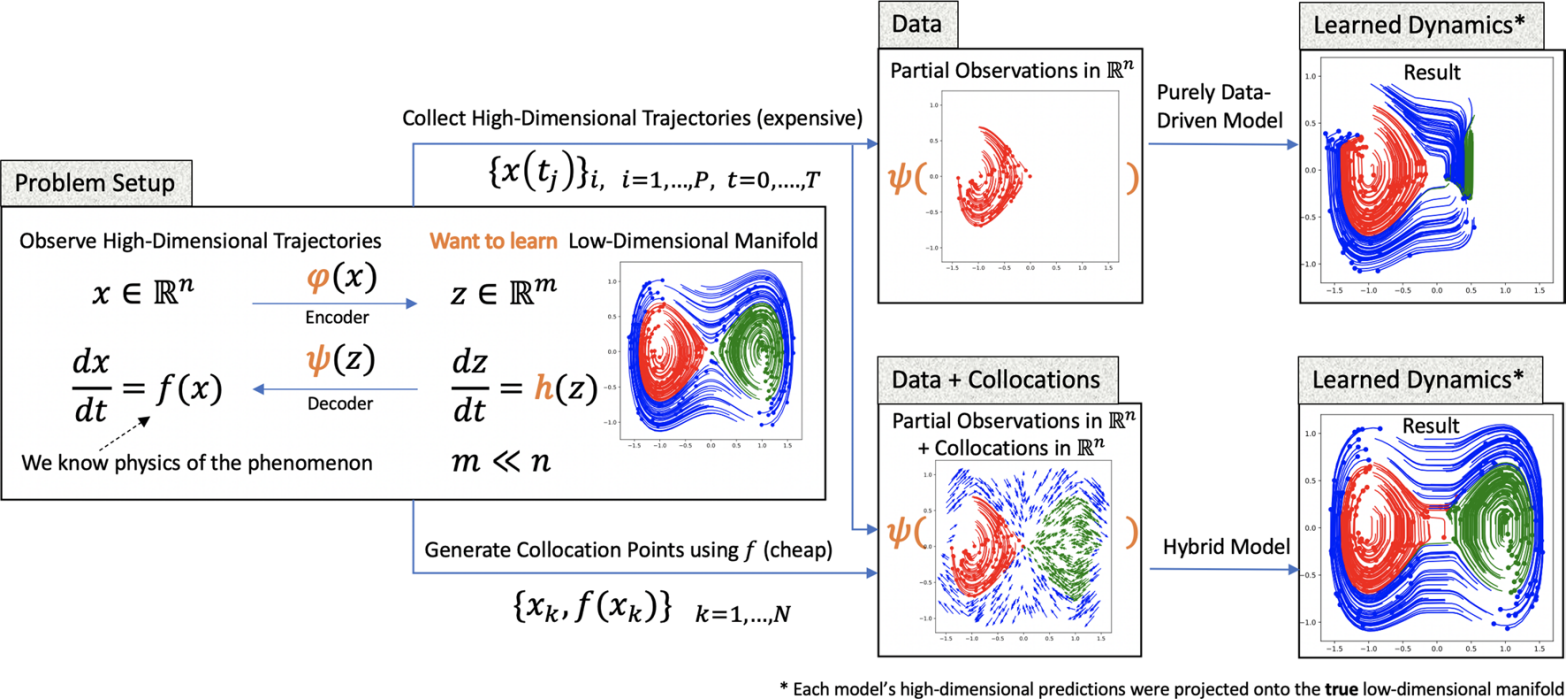
we use a toy example—a Lifted Duffing Oscillator—to show that it is possible to “fill the gaps” in data with collocation points. Specifically, the Hybrid model is able to learn the dynamics of two additional basins of attraction that were not represented in the dataset. As shown in the top-rightmost frame, without the collocation points the model does not infer the dynamics in the unseen regions correctly.

## Experiments

The experiments section is organized as follows. First, to illustrate the ideas behind the framework we study its performance on a high-dimensional ODE—a lifted Duffing oscillator. We show how a non-linear latent dynamics $\varvec{h}(\varvec{z})$ overcomes the limitations of DMD and Koopman networks from^28^ by handling multiple basins of attraction within one model. We also show that using physics-informed loss is sufficient for reconstructing the behaviour for basins of attraction that are not represented by the data. Finally, we demonstrate that a purely data-driven model may be highly-accurate in the short-term and highly unstable in the long-term, even when the data is abundant, and show that the physics-informed approach improves long-term stability of such models by multiple orders of magnitude.

Next, we study the framework’s performance on Burgers’ equation. We show that (i) the non-linear latent dynamics model yields more compact latent space representations than its linear counterpart for the same accuracy; (ii) the compact latent space representations allow for more stable long-term predictions; (iii) in the presence of significant noise in the data, the use of collocation points improves stability by providing an extra source of information that is noise-free, and (iv) in certain scenarios, training *only* on collocation points yields *better* models than training on data, even when a vast amount of data is available. The last observation shows that the contribution of the physics-informed loss (9) may surpass that of the data-based loss (4), especially when the data is severely limited or noisy.

### Lifted duffing oscillator

A Duffing oscillator is a dynamical system $d\varvec{z}/dt = \varvec{h}(\varvec{z})$ such that

11 $$\begin{aligned} \begin{aligned} \frac{dz_1}{dt}&= z_2 \\ \frac{dz_2}{dt}&= z_1 - z_1^3. \end{aligned} \end{aligned}$$

**Figure 4:**
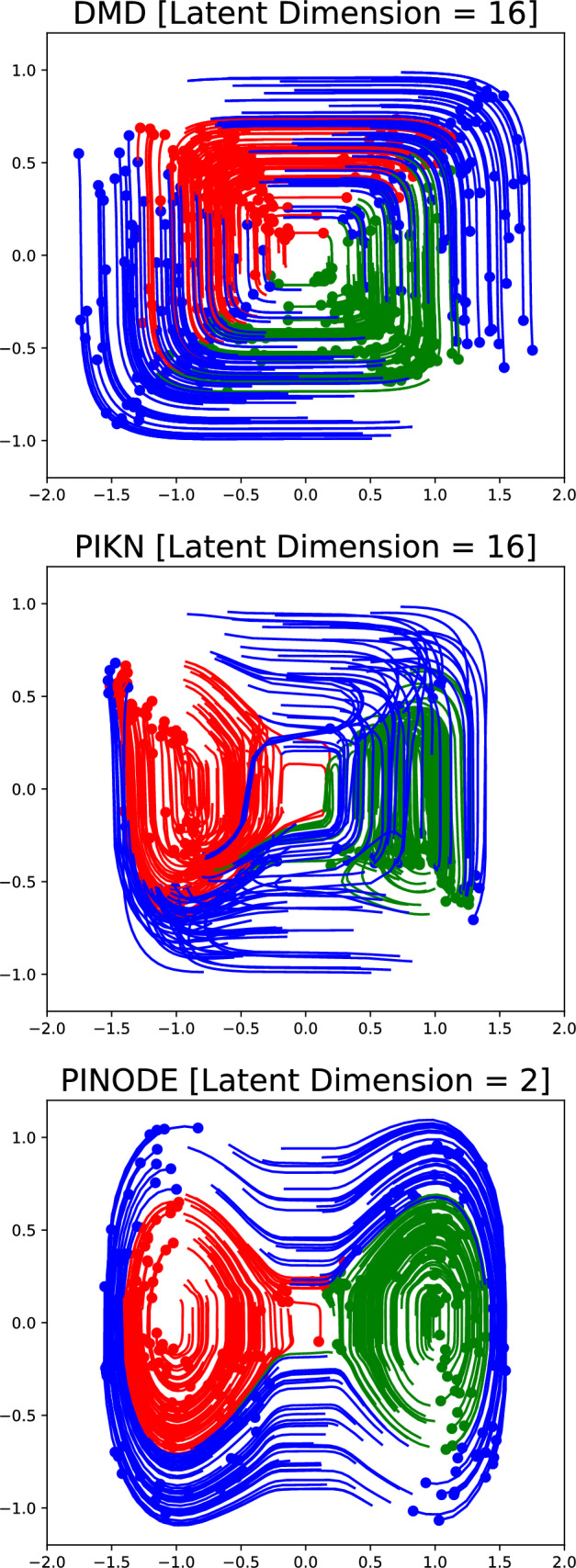
Non-linearity in the latent dynamics and the autoencoder employed in teh PINODE Hybrid model are important for accurate long-term extrapolation. The DMD model and PIKN Hybrid model were unable to extrapolate the dynamics from collocation points.

A phase portrait for 300 randomly sampled trajectories from this system is visualized on Fig. 3, left frame. Depending on the total energy, each trajectory always stays in one of three regions: the left lobe, the right lobe, or the outer area, visualized in red, green, and blue, respectively. To create a synthetic high-dimensional system that retains this property, we lift the Duffing trajectories into a higher-dimensional space by applying an invertible transformation $\mathscr {A}(\varvec{z})$:

12 $$\begin{aligned} \varvec{x}:= \mathscr {A}(\varvec{z}) = A\varvec{z}^3, \quad A \in {\mathbb {R}}^{128 \times 2}, \quad A_{ij} \sim _{i.i.d.} \mathscr {N}(0, 1). \end{aligned}$$

Hence, for this system $z \in \mathscr {Z}= {\mathbb {R}}^2$ and $\varvec{x} \in \mathscr {X}= \text {span}\{A_{:,1}, A_{:,2}\} \subseteq {\mathbb {R}}^{128}$. We treat $\mathscr {X}$ as an observable space, in which the dynamical system (11) obeys the following:

13 $$\begin{aligned} \frac{d\varvec{x}}{dt} = \varvec{f}(\varvec{x}) = \nabla ((A^TA)^{-1}A^T\varvec{x}^{1/3})^T\varvec{h}((A^TA)^{-1}A^T\varvec{x}^{1/3}). \end{aligned}$$

Thus, we created a high-dimensional dynamical system with multiple basins of attraction for which the dynamics $\varvec{f}$ are known.

For the experiment, we generate 6144 trajectories $\varvec{x}_i$, $t=[0, 1]$, $\Delta t = 0.1$, all taken from the left lobe region (in red). We also sample 50,000 collocation points $\bar{\varvec{x}}_j$ from the right (green) and the outer (blue) regions each by sampling $\bar{\varvec{z}}_j \in U\left( [-3/2,\, 3/2] \times [-1, 1]\right)$ and then applying the transformation (12). For this example the conditions for collocation points discussed in “Methods” section are trivially satisfied.

We train two PINODE models: a Data-Driven model that only uses the trajectories, and a Hybrid model that uses both trajectories and collocation points. The models share the same architecture and training parameters that are detailed in Supplementary Appendix A.1. After training, we invert the mapping (12) to project the models’ high-dimensional predictions for unseen initial conditions onto the true low-dimensional manifold; those are visualized in Fig. 3.

We make two observations from the results displayed in Fig. 3. First, a purely data-driven model is unable to extrapolate outside its training region using only the data from that region. This observation is consistent with the conclusions from related works^17^ that neural networks interpolate well but struggle with extrapolation tasks. Second, we see that collocation points provided enough extra information for the model to predict nearly perfectly in regions from which no trajectories were provided. This observation suggests that one can use collocation points to “cover the gaps” in data and improve the extrapolation accuracy of the model.

**Figure 5:**
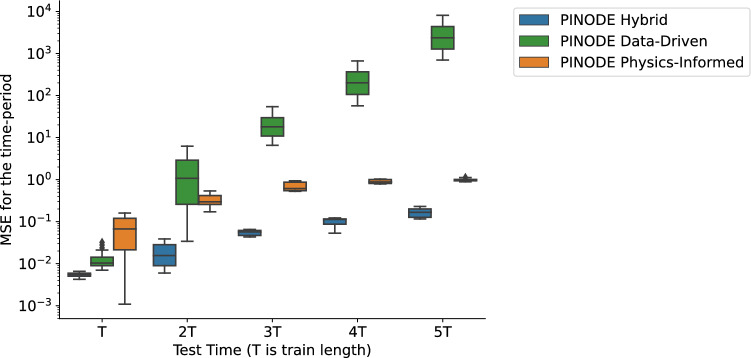
Box plots of the prediction error for three PINODE models: Data-Driven, Physics-Informed, and Hybrid. The time is measured in multiples of the training time period, i.e. $x=3T$ refers the time-range between two and three training time-periods away.

The ability of Neural ODE to model nonlinear dynamics in the latent space is demonstrated in Fig. 4. The figure shows a comparison between the Hybrid PINODE model, the Hybrid PIKN model^28^, and DMD, all of which have been trained using the same dataset. PIKN differs from PINODE in that it uses linear latent dynamics $\frac{dz}{dt} = Lz$, where *L* is a finite-dimensional approximation of the Koopman operator, instead of a general non-linear dynamics operator $\frac{dz}{dt} = h_\theta (z)$. For PIKN, we set $z \in {\mathbb {R}}^{16}$, an 8 times expansion of the dimension of the true manifold. We observe in Fig. 4 that PIKN is unable to extrapolate the dynamics to unseen areas correctly using the collocation points: eventually, all trajectories “collapse” onto the same attractor. It can also be seen that DMD shows even worse performance which could be attributed to its linear model reduction.

In the next experiment, we show that collocation points stabilize long-term predictions of the model even when data from all parts of the space are available. To illustrate, we generate a dataset of 6144 trajectories (2048 trajectories per red, green, and blue area) and 50,000 collocation points uniformly distributed among all three lobes. We train three models: Data-Driven, Physics-Informed, and Hybrid versions of PINODE. The relative performance of the three models is evaluated in Fig. 5, where the x-axis represents the test time-horizon as multiples of the training trajectory length *T*. The *y*-axis shows box plots of the prediction mean squared error (MSE) corresponding to 300 unseen trajectories within the specific period. For example, $x = 2T$ represents the time-period [2*T*, 3*T*), and the *y*-axis shows the distribution of the prediction errors within the period [2*T*, 3*T*). Figure 5 shows that the performance of the Data-Driven model degrades quickly when the forecasting time-period increases despite its excellent performance when forecasting within its training time-period. The Physics-Informed model starts with modest performance over the training time horizon but maintains a stable performance when forecasting far ahead. The Hybrid model, in its turn, combines both near-term accuracy with long-term stability, yielding the best results over each time period.

### Burgers’ equation

We now study the performance of our framework on Burgers’ equation with $[-\pi , \pi ]$-periodic boundary conditions:

14 $$\begin{aligned} {} & u_t + uu_x = \nu u_{xx} \\&u(-\pi , t) = u(\pi , t),\quad \forall t \in [0, T], \end{aligned}$$

where $u_t$, $u_x$, and $u_{xx}$ represent partial derivatives in time, the first, and second spatial derivatives, respectively. Burgers’ equation is a PDE occurring in applications in acoustics, gas and fluid dynamics, and traffic flows^42^. When $\nu$ is significantly smaller than one, the system exhibits strong non-linear behaviour and is called “advection-dominated”, otherwise when $\nu$ is large the system is called “diffusion-dominated”. In the case of the former, linear projection methods such as POD become inaccurate as the true solution space has a slow decaying Kolmogorov n-width, manifesting itself in slow decaying singular values^43^. Therefore, in this section we focus on the advection-dominated Burgers’ equation for which we set $\nu = 0.01$.

To generate trajectories, we discretize the spatial domain $[-\pi ,\,\pi ]$ into 128 grid-points, and solve Eq. (14) for $t \in [0, 2]$ with $\Delta t = 0.1$ using a spectral solver^44^. To generate a diverse set of initial conditions we sum the first 10 harmonic terms with random coefficients:

15 $$\begin{aligned} u(x, 0) = \frac{1}{10}\sum _{k = 1}^{10} a_k\cos (kx) + b_k\sin ((k+1)x), \quad a_k, b_k \sim \mathscr {N}(0, 1). \end{aligned}$$

To generate collocation points we use the same family of functions as we used for the initial conditions in Eq. (15), and additionally randomize the presence of individual frequencies in the sum:

16 $$\begin{aligned} \bar{u}(x) = \frac{1}{10}\sum _{k = 1}^{10} p_ka_k\cos (kx) + q_kb_k\sin ((k+1)x), \quad a_k, b_k \sim \mathscr {N}(0, 1), \quad p_k, q_k \sim Be(1/2). \end{aligned}$$

We choose this family of collocation points to meet the conditions (2.5). First, this family is representative of the state space $\mathscr {X}\times Im_f(\mathscr {X})$ in the region of interest (moving wave-fronts). Second, (16) is a smooth set of functions that does not contain unattainable states. Finally, and more importantly, the values $u_x$ and $u_{xx}$ and, consequently $u_t$ can be computed analytically, which makes it especially cheap to sample large numbers of collocation points.

### Compressibility of the latent space

In “Lifted duffing oscillator” section, we showed that a non-linear finite-dimensional latent dynamics model can be necessary for building a compact ROM for the high-dimensional lifted Duffing system. That is *not* necessarily the case for Burgers’ equation since there exists the Cole-Hopf transformation that linearizes the dynamics for Burgers’ equation. However, a latent-space non-linearity can, in principle, be utilized for finding a more compact latent space representation, or for increasing the forecast accuracy for a fixed latent space dimension. In this section, we demonstrate how PINODE can achieve both goals.

**Figure 6:**
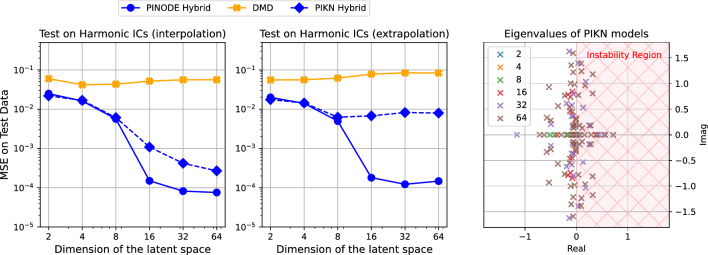
PINODE Hybrid model utilized the latent space dimension 5 times more efficiently in terms of MSE than PIKN Hybrid model when modelling low-viscosity (highly-nonlinear) Burgers’ equation (left frame). The difference in performance grows to $\times$ 100, when forecasting two times farther than the training period (central frame). PIKN suffers from long-term instability due to the presence of eigenvalues with positive real part in the latent dynamics matrix (right frame). In this frame we plot all the eigenvalues of the latent-space matrix for each PIKN model from frames 1 to 2. The legend in the right frame refers to the dimension of the latent space used by the corresponding PIKN model.

For this experiment we generate 16,384 trajectories as described in (15). We also generate 100,000 collocation points as described in (16). The purpose of using such a large amount of data is to allow the trained models to achieve the best performance for the specified latent space dimension. We evaluate the performance of the models on test data with two different time-frames: (1) same as that of training data (*interpolation*), and (2) two times longer than that of the training data (*extrapolation*). More details on the experimental setup are provided in Supplementary Appendix A.4.

In Fig. 6, we compare the performance of the three models: DMD, PIKN Hybrid, and PINODE Hybrid. First, we notice that DMD does not perform well on the test data, despite achieving a training loss ($\sim 10^{-3}$). This observation is consistent with earlier works^8,45^; and illustrates well that a combination of a linear encoder and a linear latent dynamics operator may not be sufficient for modelling highly-nonlinear phenomena. Second, we notice that PINODE achieves better performance for a given latent space dimension compared to PIKN. For instance, for $m = 16$ (Fig. 6, left pane), PINODE achieves $\sim 5$ times lower mean squared error than PIKN, which achieves the same performance only when $m = 512$. More importantly, PINODE maintains a low prediction error over a longer-term horizon (extrapolation in time), which is not the case for PIKN (Fig. 6, center pane). This is a consequence of the latent-dynamics matrix ($h(z) = Lz$) of PIKN having eigenvalues with positive real parts, which implies long-term instability (Fig. 6, right pane). Although there has been progress in the literature^46^, further research is needed to understand (i) how to enforce stability constraints for PIKN, and (ii) why one does not need the same enforcement for PINODE to exhibit stable behaviour.

### Training in low-data regime with collocation points

In the next experiment, we study the relative efficiency of using collocation points against using data in a low-data regime. It is frequently the case that only a small number of simulations (or measurements) can be obtained for a physical system of interest due to the computational, time, or budget constraints. We would like to compensate the lack of sufficient data with providing collocation points which are considerably cheaper to generate. In this section, we show that, when chosen appropriately, collocation points can be effectively used for training a model in the low-data regime, and their contribution to a model’s accuracy may even surpass the contribution of the data.

**Figure 7:**
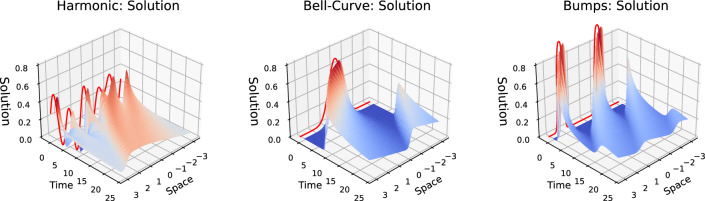
Examples of “harmonic”, “bell-curve”, and “bump” initial conditions, as well as the resulting solutions, in columns 1, 2, and 3, respectively.

**Figure 8:**
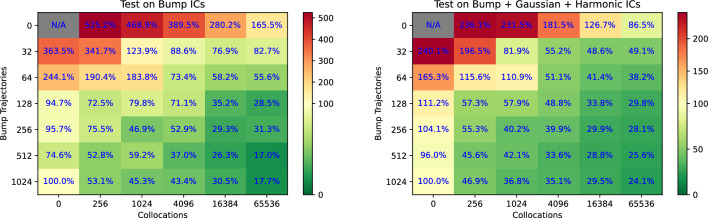
Comparison of the achievable MSE relative to the full data regime (1024 trajectories). When the data is scarce, collocations-based physics-informed loss improves the forecasting accuracy of ROMs by an average of 5 times lower MSE compared to the data-only regime, as shown in this experiment with Burgers’ equation. When other types of initial conditions (“harmonic”, “bell-curve”) are used, the physics-only model (top-right corner of the right frame) outperformed the most data-rich model in our experiment (bottom-left corner).

To illustrate the trade-off between data and collocations, we train one model using varying combinations of the number of trajectories vs collocation points in their training datasets. To gauge the extrapolation power of our models, we use trajectories with three types of initial conditions: “harmonic”, “bell-curve”, and “bumps” (see Fig. 7 for illustrations). We generate 1024 trajectories with “bumps” initial conditions for the training data, and use the harmonic family of initial conditions as described in (16) for generating the training collocations. We use two test datasets: (1) 100 trajectories with “bump” ICs to assess within-distributuion performance, left frame), and (2) a mix of trajectories with “bump”, “bell-curve”, and “harmonic” initial conditions, 100 trajectories each, to assess out-of-distribution performance. All test data trajectories are two times longer than the training trajectories. More details on the experimental setup are provided in Supplementary Appendix A.5. Figure 8 presents the reconstruction MSE of the test datasets obtained from a PINODE models that were trained on varying combinations of trajectories and collocation points as a percentage of the MSE achievable by a PINODE model that was trained on the full 1024 trajectories alone (no collocations). The PINODE models all use a latent space dimension $m=16$.

Figure 8 demonstrates that adding collocation points consistently improves the model performance in our experiments. Moreover, when a sufficient number of collocation points is added in training, the model with fewer training trajectories was always able to outperform the model that was trained on all the available trajectories and no collocations. On average, a collocation-aided model was *5 times better* at both within-distribution and out-of-distribution reconstruction relative to a purely data-driven version of the model. In addition, we noticed that a model that used only collocation points can perform better than a data-rich model, especially when predicting the dynamics of the unseen initial conditions (Fig. 8, right pane, top-right vs bottom-left corner).

**Figure 9:**
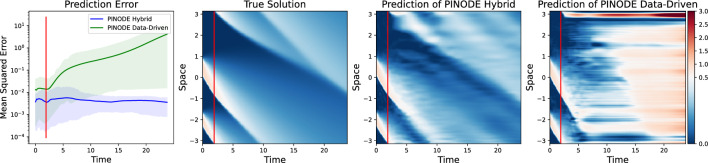
The first subplot shows the relative error of solving Burgers’ equations on 100 test (unseen) initial conditions for two models: PINODE Hybrid and PINODE Data-Driven. Both models interpolate well but a purely data-driven model fails to extrapolate past the training time-horizon (left of the red vertical line). PINODE-Hybrid provides stable long-term predictions that points to its ability to correctly discover the low-dimensional manifold dynamics.

We also notice that the Hybrid models yield more stable and accurate predictions, relative to their purely data-driven counterparts, when forecasting far beyond the training time-period. In Fig. 9 we visualize the predictions for a test IC for two models: Data-Driven model from the bottom-left corner of Fig. 8, and a Hybrid model from the bottom-right corner of Fig. 8. The red line separates the time-period of training from the time-period of forecasting. The hybrid model’s errors stay below $10^{-2}$ even when forecasting 10 times farther than what it was trained on. In contrast, the Data-Driven model shows low errors within its training time-region but the forecast errors grow quickly when forecasting beyond that.

**Figure 10:**
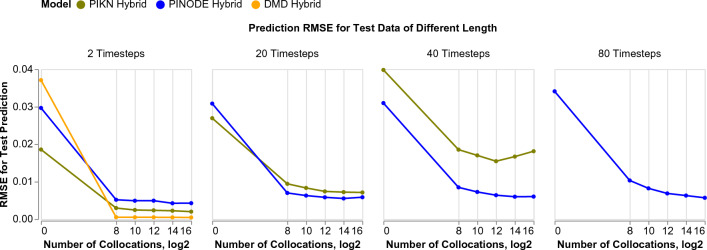
Collocation points improve results of all three models but they don’t fix models’ inherent shortcomings like instabilities in linear latent dynamics.

Finally, we observe that using collocation points can benefit other models, like DMD and PIKN. To illustrate, we replicate the experiments from Fig. 8 where the number of trajectories is 256 and with Bump ICs for PINODE, PIKN, and DMD. Figure 10 shows the root mean squared error (RMSE) for the test data predictions as a function of the number of collocation points that were used in training. The figure illustrates the prediction error for increasing prediction horizons going from left to right, and demonstrates that in all cases, PINODE benefits from the available collocation points. The leftmost panel shows that every model improves its one-step-ahead predictions, with DMD quickly achieving near-optimal performance. However, once the forecast horizon is increased to 20 timesteps ahead (length of the training trajectories) and above, DMD failed to correctly forecast the long-term trajectories and was removed from those figure to improve legibility. The PIKN models improved the one-step-ahead (1st pane) and interpolation performance (2nd pane) by a factor of 4. It also improved the extrapolation performance for 40-steps prediction (3rd pane) but failed to extrapolate for 80 steps (4th pane, removed for legibility). We attribute this behavior of PIKN to the possibility that the latent dynamics operator of PIKN contains positive eigenvalues despite the use of collocation points.

### Robustness to noise in the low-data regime

In this section we show that the use of collocation points improves the ROMs’ robustness to noise in the data by providing an alternative, noise-free, source of information.

**Figure 11:**
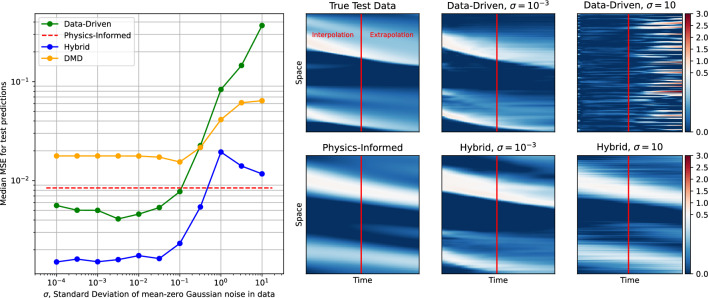
Physics-informed loss works as a safeguard that prevents unbounded performance drop when quality of the data degrades due to noise. Namely, the solution of the hybrid loss (10) converges to the solution of the physics-informed loss (9), when the data-driven loss (9) becomes uninformative. The performance of purely data-driven methods (Data-Driven, DMD) grows unbounded since these models don’t have an alternative noise-independent source of information.

For this experiment, we use the Burgers’ equation dataset containing 1024 trajectories with “bump” initial conditions, and 65,536 “harmonic” collocation points as defined in Eq. (16). We then add i.i.d. Gaussian noise to the trajectories, with variance ranging from $\sigma = 10^{-4}$ to $\sigma = 10$. For reference, most of the data values lie between 0 and 1, so a noise level with $\sigma > 1$ dominates the data. We train four models: PINODE Hybrid, PINODE Data-Driven, PINODE Physics-Informed, and DMD. To measure the models’ out-of-distribution prediction errors, we use the test dataset with Bump, Gaussian, and Harmonic initial conditions, as described in the previous subsection. The prediction errors are displayed in Fig. 11, left pane. The prediction error of a purely Physics-Informed model (in red) is flat because the collocation points are noise-free.

Figure 11 shows that in the high noise setting, the error of purely data-driven models (DMD and PINODE Data-Driven) grows unbounded, whereas the performance of the hybrid model converges to the performance of the Physics-Informed model as the noise level increases. We hypothesise that such behavior is due to the second part ($\mathscr {L}_{\theta }^{data}$) of the combined loss (Eq. 10) turns into noise, and so its derivative also turns into noise.

17 $$\begin{aligned} \nabla \mathscr {L}_{\theta } = \underbrace{\nabla \mathscr {L}_{\theta }^{physics}}_{\text {informative}} + \underbrace{\nabla \mathscr {L}_{\theta }^{data}}_{\text {noise}}. \end{aligned}$$

Thus, one can think about optimizing a hybrid model (10) as about training a Physics-Informed model (9) using a noisy gradient descent with a fixed-variance noise. From the optimization literature^47–49^ we know that, under certain conditions, such SGD converges to a neighbourhood of a local minimum of its loss (in this case $\mathscr {L}_{\theta }^{physics}$) with high probability. So instead of diverging, a hybrid model turns into a Physics-Informed model; where the latter works as a performance safeguard in the high-noise regime. On the right hand-side of Fig. 11, we show an example of the prediction performance of each of the models described above. The data-driven and hybrid models yield visually similar solutions when $\sigma = 10^{-3}$. However, the former provides inadequate performance when the data is dominated by noise, whereas a hybrid model in this regime produces a solution that is visually similar to the one that the Physics-Informed model produces. A more rigorous analysis of this phenomenon seems possible but lies outside of the scope of this paper.

## Discussion and conclusions

In this work, we demonstrated how a collocation point-based technique can improve the performance of an emerging class of continuous-time physics-informed neural-network based reduced-order models. First, we demonstrated that the incorporation of collocation points in training data can “cover the gaps” in training trajectories and inform the model about underrepresented basins of attraction. Such an approach alleviates the demand for large volumes of data that is common in network-based models, which is crucial in applications where data is scarce and expensive. Second, the physics-informed loss may work as a safeguard, providing a noise-free source of underlying dynamics. Third, collocation points can stabilize the model’s long-term predictions, allowing for accurate forecasting far beyond the training time horizon. Finally, together with using a NODE-based non-linear latent dynamics, adding physics-informed loss leads to the discovery of more compact latent space representations that also yield more accurate models. Simultaneous stability and compactness is especially important if one aims to use models together with compressive sensing and control algorithms. With respect to the computational complexity, we note that adding *Tk* collocation points to the training imposes less of a computational burden than adding *k* data trajectories because collocation points do not require computing integrals forward in time as in the case of data trajectories.

One clear limitation of the current work is that the choice of an efficient collocation family is a design decision that a practitioner makes. The authors believe that such decisions can be automated by adopting existing approaches from classic works on numerical approximations of PDEs, which we leave for future research. Another automation that prompts future research is deriving efficient ways of sampling collocation points, possibly via applying modern adaptive learning techniques^50^. Finally, although “Robustness to noise in the low-data regime” section provides some rationale for why one may expect robustness of Hybrid models under noise, the authors believe that a more rigorous analysis is possible; particularly one that provides conditions under which such robustness is guaranteed.

## Supplementary Information

Supplementary Information.

## Data Availability

The datasets used and/or analysed during the current study available from the corresponding author on reasonable request.
