# Supplementary material for: Physics-informed neural ODE (PINODE): embedding physics into models using collocation points
Source: Sci Rep. 2023 Jun 22;13:10166. doi: 10.1038/s41598-023-36799-6 (PMC10287651; doi:10.1038/s41598-023-36799-6)
Supplement: Supplementary file 1 — Supplementary Information. [file 41598_2023_36799_MOESM1_ESM.pdf]

A Details for Experiments Design

Hardware All experiments were computed using an `Intel(R) Xeon(R) CPU E5-2630 v4 @ 2.20 GHz` equipped with a `Tesla K80` GPU. The computer had `Linux 4.15` installed as an OS.

A.1 Lifted Duffing Oscillator: Learning Unseen Basins with Collocation Points (Figure 3)

In this experiment we trained two models; both share the same architecture but differ in the input data, as described in Chapter 3.1.

Architecture The network consists of two blocks: the autoencoder pair $\phi_\theta(x)$, $\psi_\theta(z)$, and the latent dynamics $h_\theta(z)$, where θ represents the combined weights of all networks.

Both ϕ and ψ were fully-connected networks with three layers, the input-output dimension of 128, the bottleneck-space dimension of 2, and the hidden-layer dimensions of 256. The hidden layers had ReLU activations except for the output layers of ϕ and ψ which had linear activation.

The network h was a fully-connected network with three layers, with the input and output dimension of 2 and the hidden-layer dimensions of 128. All hidden layers had ReLU activation, the output layer had a linear activation.

We used standard network classes of `pytorch`[2] by[51] to implement the networks, and we used a differentiable integrator `torchdiffeq`[3] by[30] for evaluating derivatives of the loss function.

Data For data **snapshots** we used 6144 trajectories 10 steps long each, with a step-size $dt = 0.1$. For **collocation points** we generated a set of 10^5 2-dimensional points $\bar{z}_j \in U([-3/2, 3/2] \times [-1, 1])$, excluded those belonging to the left (red) lobe, and projected them to the observable space $\mathscr{X}$ using the true decoder (12). We then used those high-dimensional points $\bar{x}_j$ as collocation points.

Training We trained the combined model for 400 epochs, with the learning rate of 10^{-4}. All weights ω_i were set to 1 for a hybrid model, and ω_2, ω_4 were set to 0 for a data-driven model.

In each batch we had 64 trajectories and, in case of hybrid models, 640 collocation points. The rationale behind this ratio is that one trajectory contains number-of-steps snapshots of the system. Hence, to balance the amount of information that comes from both sources within a batch, we were taking the number-of-steps more collocation points than trajectories for every batch. The same rationale holds for all other instances of training of a hybrid model in this paper.

A.2 Lifted Duffing Oscillator: Far-Out Forecasting (Figure 5).

In this experiment we trained three models, all sharing the same architecture but differ in their loss functions, namely in the coefficients w_i.

Architecture The networks architectures match to the one described in Appendix A.1.

Data For data **snapshots** we used 6144 trajectories (2048 for each of the attractors) 10 steps long each, with a step-size $dt = 0.1$. For **collocation points** we generated a set of 10^5 2-dimensional points $\bar{z}_j \in U([-3/2, 3/2] \times [-1, 1])$ and projected them to the observable space $\mathscr{X}$ using the true decoder (12). We then used those high-dimensional points $\bar{x}_j$ as collocation points.

Training We trained the combined model for 400 epochs, with the learning rate of 10^{-4}. All weights ω_i were set to 1 for a hybrid model, ω_3, ω_4 were set to 0 for a data-driven model, and $\omega_1 = \omega_2 = 0$ for Physics-Informed model.

A.3 Lifted Duffing Oscillator: Role of Non-Linear Latent Dynamics (Figure 4).

In this experiment we trained three models: DMD, PIKN, and PINODE.

Architecture The PINODE model's architecture matches to the one described in Appendix A.1. The PIKN model's architecture is the same except that the latent dynamics $h(z) = Lz$ is linear: it consists of one fully-connected linear layer of width 16 with no bias and no activation function. DMD model had the latent space of 16; the implementation is faithful to the original works[8].

Data The datasets for both trajectories and collocation points match to the ones described in Appendix A.2

Training We trained the combined model for 400 epochs, with the learning rate of 10^{-4}. All weights ω_i were set to 1 for both PIKN and PINODE. DMD model used no collocation points, whereas PIKN and PINODE used both trajectories and collocation points.

[2] https://pytorch.org
[3] https://github.com/rtqichen/torchdiffeq

A.4 Burgers' Equation: Compressibility (Figure 6)

In this section we study how efficiently different ROMs use the same size of the latent space.

Architecture In **PINODE** and **PIKN** models, both ϕ and ψ were fully-connected networks with three layers, the input-output dimension of 128 and the hidden-layer dimensions of 512. The hidden layers had ReLU activation except for the output layers of ϕ and ψ which had linear activation. The size of the latent space was varying from 2 to 512, see the x-axis of Figure (6). In **PINODE**, the network h was a fully-connected network with three layers with the hidden-layer dimensions of 512. All hidden layers had ReLU activation, the output layer had a linear activation. For **PIKN** the network had one layer of the latent space size with no bias and linear activation. In other words, $h(z) = Az$. For **DMD** we used a classic algorithm from[8], and set the number of DMD modes to be equal to the size of the latent dimension.

Data For **snapshots**, we generated 16384 trajectories of the system for our train dataset and 300 trajectories for our test dataset. Each trajectory had 40 time-steps with $dt = 0.1$. We used randomly-generated functions from Equation 15 as initial conditions for the trajectories. We used only first 20 time-steps of train trajectories for training. We used the first 20 steps of the test trajectories to evaluate *interpolation* performance of the model, and the next 20 time-steps for evaluating *extrapolation* performance. All performance measures on Figure (A.4) are based on the test dataset. For **collocation points**, we used Equation (16) to generate 10^5 collocation points.

Training We trained models for 500 epochs, with the learning rate of 10^-4. All weights ω_i were set to 1 for a hybrid model, and ω_3, ω_4 were set to 0 for a data-driven model. Every batch contained 64 trajectories and 1280 (that is, 64*20) collocation points, with the same rationale as in Appendix A.1.

A.5 Burgers' Equation: Data-vs-Collocations (Figure 8)

In this section we study the relative impact of data and collocation points as training datasets to the performance of the resulting models.

Architecture In **PINODE** models both ϕ and ψ were fully-connected networks with three layers, the input-output dimension of 128, the latent-space dimension of 16 and the hidden-layer dimensions of 512. The hidden layers had ReLU activation except for the output layers of ϕ and ψ which had linear activation. The network h was a fully-connected network with three layers with the input-output dimension of 16, and the hidden-layer dimensions of 512. All hidden layers had ReLU activation, the output layer had a linear activation.

Data For **snapshots**, we generated 2048 trajectories of the system for our train dataset. Each trajectory had 20 time-steps with $dt = 0.1$. We used randomly-generated functions from Equation 15 as initial conditions for the train trajectories. For **collocation points**, we used Equation (16) to generate 65536 collocation points. We generated 300 trajectories for our test datasets, 100 per kind of initial conditions from Figure (7). We used all 40 steps of the test trajectories to evaluate the performance of the models. All performance measures on Figure (A.5) are based on this test dataset.

Training We trained models for 500 epochs, with the learning rate of 10^-4. All weights ω_i were set to 1 for a hybrid model, and ω_3, ω_4 were set to 0 for a data-driven model. Every batch contained 64 trajectories and 1280 (that is, 64*20) collocation points, with the same rationale as in Appendix A.1.

A.6 Burgers' Equation: Robustness to Noise (Figure 11)

In this section we examine robustness to noise for four models: PINODE (Data-Driven, Physics-Informed, Hybrid). We also add DMD to the comparison as a reference point.

Architecture All PINODE models share the same architecture as described in Appendix A.5.

Data We start with the same data as described in Appendix A.5. Then we apply 11 different levels of Gaussian noise with the mean 0 and the variances distributed log-uniformly between $[10^{-4}, 10^1]$. We only apply noise to the train snapshots; the test snapshots are noise-free.

Training We trained models for 500 epochs, with the learning rate of 10^-4. All weights ω_i were set to 1 for a hybrid model, ω_1, ω_2 , and ω_3, ω_4 were set to 0 for a data-driven model. Every batch contained 64 trajectories and 1280 (that is, 64*20) collocation points, with the same rationale as in Appendix A.1.
